# Food insecurity as an underexplored pathway linking ethnic enclave contexts and anxiety among Dominicans in the United States

**DOI:** 10.3389/fnut.2026.1778246

**Published:** 2026-04-28

**Authors:** Karen R. Flórez, Yuntian Bi, Emily M. D'Agostino, Khushboo R. Agarwal, Sandra S. Albrecht, Sarah Marrara, Ana F. Abraído-Lanza, Ramona Hernández

**Affiliations:** 1 Graduate School of Public Health and Health Policy (CUNY SPH), Center for Systems Science and Community Design, City University of New York, New York, NY, United States; 2 Nicholas School of the Environment, Duke University, Durham, NC, United States; 3 Department of Population Health Sciences, Duke University School of Medicine, Durham, NC, United States; 4 Duke Center for Childhood Obesity Research, Duke University School of Medicine, Durham, NC, United States; 5 Department of Orthopedic Surgery, Occupational Therapy Division, Duke University School of Medicine, Durham, NC, United States; 6 Duke Clinical Research Institute, Duke University School of Medicine, Durham, NC, United States; 7 Duke Global Health Institute, Duke University School of Medicine, Durham, NC, United States; 8 Graduate School of Public Health and Health Policy (CUNY SPH), Epidemiology and Biostatistics, City University of New York, New York, NY, United States; 9 Department of Epidemiology, Columbia University Mailman School of Public Health, New York, NY, United States; 10 School of Social Work, Columbia University, New York, NY, United States; 11 City College of New York, CUNY Dominican Studies Institute, New York, NY, United States

**Keywords:** anxiety, Dominican immigrants, food insecurity, neighborhoods, residential segregation

## Abstract

**Introduction:**

Migration shapes access to food environments and practices, with implications for both nutritional and mental health. Although neighborhood ethnic concentration has been linked to mental health among Latino populations, the findings remain inconsistent, and the mechanisms are not well understood. Food insecurity, a common experience among immigrant households, may represent a key psychosocial pathway linking the neighborhood context to anxiety. Caribbean Latino populations, including Dominicans, remain underrepresented in this literature. This study examined whether Dominican ethnic concentration is associated with anxiety symptoms and whether household food insecurity mediates this relationship.

**Methods:**

We analyzed data from a representative multi-state sample of Dominican adults in the United States (*n* = 662), 53% of whom were immigrants. Dominican ethnic concentration was operationalized using location quotients at the ZIP Code Tabulation Area level to capture enclave-like contexts in the data. Household food insecurity was assessed using a validated measure (low/very low; mild/high), and anxiety symptoms were measured using the Generalized Anxiety Disorder−4 scale (0 = not at all to 4 = nearly every day). Survey-weighted generalized structural equation models were used to test mediation, adjusting for age, sex, income, nativity, state of residence, and presence of children in the household.

**Results:**

Nearly 30% of the respondents experienced low household food security. Higher Dominican ethnic concentration was associated with lower anxiety symptoms overall, with mean GAD-4 scores being highest in low-concentration areas and lower in higher-concentration and ethnic enclaves. In survey-weighted generalized structural equation models, low household food security was strongly associated with higher anxiety symptoms (β = 0.586, *P* < 0.001). Mediation analysis indicated that household food security partially mediated the association between Dominican concentration and anxiety (*p* = 0.034).

**Discussion:**

Greater Dominican concentration may protect against anxiety by reducing household food insecurity, highlighting food insecurity as a key pathway linking ethnic concentration to mental health. These findings underscore the importance of culturally sustaining food environments for the wellbeing of immigrants and subsequent generations born in the US.

## 1 Introduction

Research on cardiometabolic risk among Latinos in the United States frequently implicates dietary changes following migration (1), often described as a “nutritional transition” from nutrient-dense traditional diets toward diets higher in calories, fat, sugar, and processed foods (2). This shift is attributed, in part, to increased exposure to obesogenic food environments in immigrant settlement contexts that lack culturally relevant food (3, 4). However, a growing body of scholarship has problematized linear and deficit-oriented narratives of dietary acculturation, noting that immigrants do not uniformly abandon traditional food practices, nor are dietary changes driven solely by a lack of access to culturally relevant foods (5, 6). Purchasing power and social position also shape food choices; for example, Latinos with a higher socioeconomic status may consume more meals from fast-food and pizza establishments than their lower-income counterparts (7). Together, this evidence suggests that dietary changes among immigrants are neither zero-sum nor uniform but rather context-dependent and socially patterned.

One critical yet underexplored dimension of these dietary transformations is food insecurity, particularly as it unfolds across migration trajectories and settlement contexts (8). For first-generation immigrants, structural constraints, such as pressure to send remittances (9), labor market exclusion (10), housing instability (11, 12), legal precarity (13, 14), and rurality (15) increase the risk of limited or uncertain access to food. Furthermore, food insecurity is linked to reduced dietary quality and increased reliance on inexpensive, shelf-stable, and calorie-dense foods (16). However, immigrant Latino groups experiencing food insecurity often consume fewer ultra-processed foods, such as sugar-sweetened beverages, than their U.S.-born counterparts, suggesting that traditional food-related norms and practices may persist despite economic constraints (17, 18). Even when dietary choices remain relatively healthful, food insecurity may exert harmful effects through psychosocial pathways (19). Food uncertainty can activate prior experiences of scarcity or displacement, intensify moral obligations around feeding family and community members, and disrupt culturally meaningful food practices, thereby contributing to psychological distress, independent of nutritional intake (20).

Beyond individual and household-level pressures, the experience of food insecurity among immigrants is also shaped by the broader neighborhood contexts in which immigrants settle. The experience of food insecurity could also be shaped by the food environments immigrants encounter in their places of settlement (21). In large urban areas, for example, immigrant settlement often gives rise to ethnic enclaves—neighborhoods characterized by a high concentration of co-ethnics (22), dense networks of ethnic businesses (23), and structural resources (24) that provide material and emotional support. While early scholarship emphasized both the protective and constraining features of enclaves (22), public health research has examined their potential salubrious effects (24, 25). The findings remain mixed and appear to vary by ethnic subgroup, gender, and health outcomes. Specifically, although some studies report null or adverse associations between residing in ethnic enclaves and cardiometabolic and perinatal outcomes (26–29) others report mixed effects on mental health (30) and certain health behaviors, but consistent benefits for diet (25, 30–34). Research specifically focused on food security and neighborhood context has found some support that greater social capital and connectedness reduce the risk of food insecurity (35–37). Increasingly, research defines social connectedness, including social support, co-ethnic networks, and a sense of belonging, as a determinant of mental health among immigrant and non-immigrant populations (38, 39). However, this research has yet to examine how neighborhood-level characteristics specific to immigrant ethnic enclaves shape these social processes or how such contexts may simultaneously influence food security and mental health.

Despite growing evidence linking neighborhood context, food security, and mental health, existing research has paid limited attention to how these relationships vary across immigrant subgroups with distinct food practices and migration histories. Studies in public health and urban sociology suggest that neighborhood characteristics, such as access to food retailers, social cohesion, and ethnic density, can shape both food security and psychological wellbeing by influencing material access to food and the availability of social support (35–39). In immigrant neighborhoods, ethnic food retail networks may also facilitate access to culturally familiar foods, even under conditions of economic hardship, allowing households to maintain traditional dietary practices despite limited financial resources (5, 40, 41). Such contexts may partially explain the paradox observed in some immigrant communities, in which food insecurity coexists with relatively high consumption of home-cooked or culturally traditional foods. However, much of the literature relies on large national samples or focuses primarily on Mexican-origin populations (42). Dominicans, one of the largest Caribbean and Latino immigrant groups in the United States, represent distinct migration trajectories characterized by circular migration, transnational ties, and dense settlement in specific urban regions (43, 44). Despite persistent socioeconomic disadvantages, prior research suggests that Dominican households rely heavily on ethnic food retailers and home cooking (5). Ethnographic and historical scholarship documents how Dominican food practices are sustained through dense ethnic food retail networks and how *comidas* function as acts of care, resistance, and belonging (45, 46), which could be related to social connectedness and mental health (38). Moreover, Dominicans often reside in immigrant neighborhoods where, despite an elevated risk of food insecurity, access to affordable and culturally familiar foods remains relatively high (5, 40, 41). Emerging evidence from other immigrant groups suggests that such contextual factors may attenuate the relationship between food insecurity and poor mental health, particularly during periods of heightened stress, such as the COVID-19 pandemic (47).

Building on this literature, the present study examines whether living in communities with a higher Dominican ethnic concentration confers a protective effect on anxiety symptoms and whether this association operates, in part, through reduced household food insecurity. Using a representative, multistate sample of Dominican adults and a location-quotient-based measure of enclave-like concentration, we tested a mediation model. We hypothesized that Dominican concentration would be inversely associated with food insecurity, which in turn would be positively associated with anxiety, and that Dominican concentration would exert both indirect and direct effects on anxiety. By integrating food insecurity into research on ethnic concentration and mental health, this study advances the understanding of how food environments and migration-related contexts jointly shape nutritional and psychological wellbeing among understudied Caribbean immigrant populations.

## 2 Study design and participants

All individual-level data for this study were drawn from the 2021 City University of New York (CUNY) Dominican Health Survey, the only U.S. health survey conducted during the COVID-19 pandemic with a representative sample of Dominicans living in the US. Briefly, a probabilistic sample of 794 participants was derived from seven states with large Dominican populations: New York, New Jersey, Florida, Massachusetts, Pennsylvania, Rhode Island, and Connecticut. The eligibility criteria included being at least 18 years of age and either born in the Dominican Republic or self-identifying as of Dominican ancestry. Participants with missing data on key analytic variables or residing in ZIP codes without corresponding census data were excluded from the analytic sample (see Analytic Sample Description below). Each participant received a US$ 25 gift card as compensation. Data were collected between September 11 and October 5, 2021, during a period marked by continued economic disruption and transnational uncertainty associated with the COVID-19 pandemic. The survey was administered by BSP Research either online or via telephone interview in the participant's preferred language (English or Spanish). Each interview was conducted entirely in one language, and respondents did not switch between languages during the survey. All survey instruments and prompts were translated and back-translated following established procedures to ensure conceptual equivalence across the English and Spanish versions. The study was approved by the Institutional Review Board of the City University of New York (CUNY-DSI, IRB File No. 2021-0474-CCNY). Area-based measures were defined according to the American Community Survey (ACS) 2022 five-year estimates (2018, 2019, 2020, 2021, and 2022).

### 2.1 Measures

#### 2.1.1 Outcome

Anxiety Symptoms of generalized anxiety were assessed using a four-item version of the Generalized Anxiety Disorder Scale (GAD), a validated and reliable screening tool for generalized anxiety disorder (48). Items asked how often during the past 7 days participants were bothered by problems such as “being able to stop or control worrying.” Responses ranged from 0 (“not at all”) to 4 (“nearly every day”) and were averaged to create a continuous scale score (0–16). Higher scores indicate greater anxiety. The internal consistency reliability for the four items was acceptable (Cronbach's α = 0.76). Because the mean scores were right-skewed, we conducted a sensitivity analysis using a log-transformed outcome; the results were substantively unchanged, and we therefore present models using the original scale for interpretability.

### 2.2 Independent variable: Dominican ethnic enclave using location quotients (LQs)

We used ACS ZIP Code Tabulation Areas (ZCTA)-level data to derive Dominican concentration based on responses to the “Hispanic or Latino Origin” item, which identifies both Latinos and specific subgroups of Latinos, such as Dominicans. Although several methods exist to operationalize ethnic enclaves, we used LQs because they capture localized concentration at a fine spatial scale and allow for a nuanced assessment of spatial clustering at the ZCTA level. LQs compare the concentration of an ethnic group in a ZCTA to its concentration in a larger reference area (e.g., a state). The LQ for each ZCTA was calculated as follows:


$$\begin{array}{l} LQ=\frac{\frac{x_{i}}{X_{t}}}{\frac{y_{i}}{Y_{t}}}\;i=1,\ldots ,N \end{array}$$

where *x*_*i*_ and *y*_*i*_ represent the ZCTA-level Dominican population and the reference (Latino) population, in ZCTA *i*, and *X*_*t*_ and *Y*_*t*_ represent their total populations within each state (across *N* ZCTAs). An LQ of 1 reflects an even distribution; values >1 indicate overrepresentation and are commonly used as evidence of enclave formation.

To avoid oversimplifying spatial heterogeneity, we also applied a four-category LQ classification for descriptive mapping: low Dominican concentration (LQ ≤ 0.8), evenness (0.8–1.2), higher Dominican concentration (1.2–2), and Dominican ethnic enclave (>2), consistent with previous studies (49–53). For regression models, we used a continuous version, but because location quotients (LQs) range from 0 to infinity and are highly right-skewed in our data, we applied a log(x + 1) transformation to stabilize variance and reduce the influence of extreme enclave values. This transformation has been recommended for asymmetric LQ distributions and produces a more interpretable continuous measure of enclave intensity (54). We then mean-centered the log variable so that model intercepts reflected the expected outcome at the average level of Dominican concentration and improved numerical stability in the mediation model.

#### 2.2.1 Mediator: household food security

Food security was measured using two standard items adapted from the USDA food security module (e.g., “We worried whether our food would run out before we got money to buy more” and “The food that we bought just didn't last, and we didn't have money to get more”). Households were classified as having low food security if one or both responses were often true and high food security if never or sometimes true, which is consistent with USDA guidelines and previous studies using this data.

### 2.3 Covariates

Drawing on prior studies focused on the neighborhood, food security, and mental health nexus among immigrants (19, 55), we controlled for sociodemographic covariates such as age (18–29, 30–49, 50+), sex (male, female), household income (< $40,000, $40,000–$79,999, ≥$80,000), presence of children in households (any/none), state/area of residence (New York, New Jersey, Florida, and other Northeast states, including Massachusetts, Pennsylvania, Rhode Island, and Connecticut), and nativity (born in the Dominican Republic/no USA or other).

### 2.4 Analyses

All analyses accounted for the complex survey design of the 2021 CUNY Dominican Health Survey. Sampling weights were applied to reflect the age, sex, educational attainment, and nativity distributions of the Dominican population based on the 2018–2022 ACS. Of the 794 survey participants, 86 were excluded due to invalid ZIP codes and 30 due to unmatched ZCTAs, leaving 678 participants eligible for the contextual linkage analysis. An additional 16 participants (< 3% of the eligible sample) were excluded due to missing data on covariates used in the regression models. Given the small proportion of missing observations, a complete-case approach was used, yielding a final analytic sample of 662 adults.

We first generated survey-weighted descriptive statistics for Dominican concentration categories (low concentration, evenness, higher concentration, and ethnic enclave; independent variable), household food security (low, high; mediator), anxiety symptom score (outcome), and covariates (age, sex, household income, state, and nativity). Bivariate analyses were used to examine differences in anxiety symptom scores, food security, and sociodemographic characteristics across the four levels of Dominican ethnic concentration using survey-weighted tabulations and mean estimates. Statistical significance was set at *p* < 0.05. All analyses were conducted using Stata/SE 15.0's survey estimation procedures (svy prefix), specifying the final calibrated weights as probability weights. Variance estimation was performed using Taylor series linearization. Survey weights were applied consistently across descriptive statistics, bivariate analyses, and generalized structural equation models. Because the survey used a single-stage sampling design without clustering, individuals were specified as the primary sampling units.

**Figure 1:**
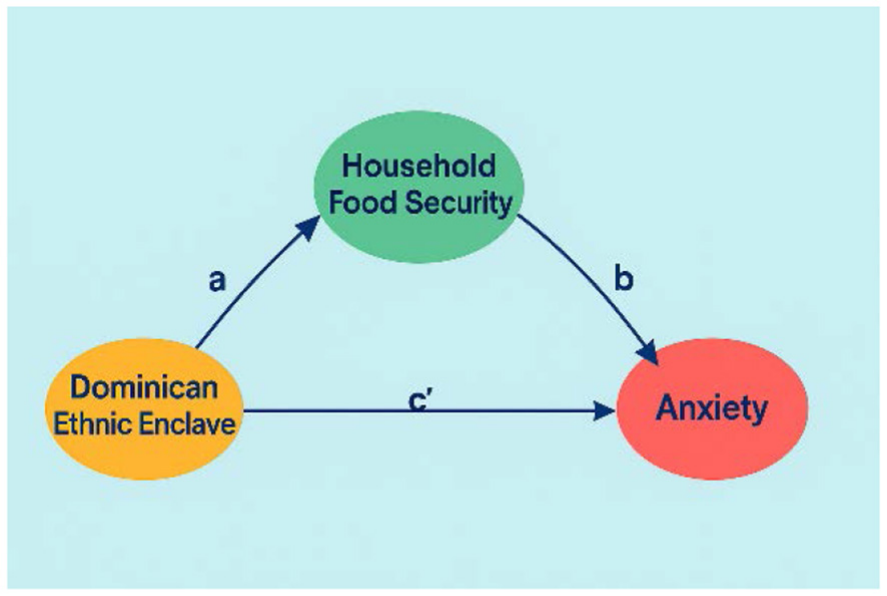
Conceptual mediation model illustrating how Dominican ethnic enclave concentration may influence anxiety symptoms indirectly through household food security and directly through other contextual mechanisms. Path a represents the association between enclave concentration and food security, path b represents the association between food security and anxiety, and path c′ represents the direct effect of enclave concentration on anxiety.

To evaluate whether household food insecurity mediates the association between Dominican ethnic enclave concentration and anxiety, we estimated survey-weighted generalized structural equation models (GSEM). Because the mediator was binary, we specified a logit model for food insecurity (path **a**) and a linear model for anxiety symptoms (path **b**), adjusting both equations for confounders at the individual level. The direct effect of Dominican ethnic concentration on anxiety (path **c′**) was estimated simultaneously. Figure 1 illustrates the conceptual mediation structure.

Indirect effects were derived as the product of paths **a** and **b**, representing the change in average anxiety symptoms associated with a one-unit increase in the LQ through its effect on the probability of being food insecure. For interpretability, we generated predicted mean anxiety scores and mean differences across the LQ categories and food security status using marginal standardization.

## 3 Results

Table 1 presents the weighted estimates for the overall sample, which was predominantly of prime working age (43.4%), with 29.0% aged 18–29 years and 27.6% aged 50 years or older. Slightly more than half of the participants were women (53.1%) and had children living in their households (52.3%). Nearly half of the respondents lived in households earning less than $40,000 annually (46.5%), and 26.7% earned $80,000 or more. New York (42.8%), Florida (21.9%), and New Jersey (18.6%) were the most common states of residence, whereas 47.2% of participants were born in the U.S. and 52.7% were born in the Dominican Republic or elsewhere.

**Table 1:** Sample Characteristics for City University of New York's (CUNY) Dominican Health Survey 2021, *N* = 662a.

Characteristic	Overall % or M (*n*)^a^	Dominican concentration^b^				*p*-value^c^
		Low concentration (*n* = 213)^d^	Evenness (*n* = 42)	Higher concentration (*n* = 80)	Ethnic enclave (*n* = 327)	
Age group						
Young (18–29)	29.01 (190)	31.86 (61)	34.19 (14)	37.66 (28)	25.21 (87)	**< 0.001**
Prime (30–49)	43.44 (323)	53.47 (125)	50.71 (22)	47.17 (41)	36.22 (135)	
Older (50+)	27.55 (149)	14.67 (27)	15.09 (6)	15.17 (11)	38.57 (105)	
Sex						
Female	53.09 (383)	51.28 (121)	52.54 (23)	49.56 (42)	54.83 (197)	0.8128
Male	46.91 (279)	48.72 (92)	47.46 (19)	50.44 (38)	45.17 (130)	
Household income						
< $40,000	46.45 (277)	35.15 (73)	37.25 (13)	43.73 (28)	54.43 (163)	**< 0.001**
$40–79k	26.82 (180)	18.59 (36)	29.03 (13)	28.87 (26)	30.86 (105)	
≥$80k	26.73 (205)	46.26 (104)	33.72 (16)	27.40 (26)	14.71 (59)	
Children in household						
No kids	47.73 (316)	33.99 (68)	52.13 (22)	51.95 (42)	61.41 (184)	**< 0.001**
Any kids	52.27 (346)	66.01 (145)	47.87 (20)	48.05 (38)	38.59 (143)	
State						
New York	42.78 (262)	39.17 (86)	34.27 (12)	19.8 (12)	50.02 (152)	**< 0.001**
New Jersey	18.56 (93)	15.83 (26)	22.32 (8)	15.67 (8)	20.2 (51)	
Florida	21.89 (214)	17.84 (56)	29.83 (17)	56.49 (55)	16.95 (86)	
Other North East (PA, MA, CT, RI)	16.76 (93)	27,15 (45)	13.58 (5)	8.05 (5)	12.83 (38)	
Nativity						
US-born	47.2 (313)	63.9 (133)	40.86 (18)	52.75 (40)	37.63 (122)	**< 0.001**
Immigrant^e^	52.72 (349)	36.1 (80)	59.14 (24)	48.25 (40)	62.37 (205)	
Food security^f^						
High/marginal	70.52 (461)	61.02 (129)	73.49 (31)	78.57 (64)	74.1 (237)	**0.008**
Low food security	29.48 (201)	38.98 (84)	26.11 (11)	21.43 (16)	25.9 (90)	
**Anxiety symptoms**	0.97 (0.03)	1.23 (0.06)	1.06 (0.13)	0.85 (0.10)	0.85 (0.05)	**< 0.001**

^a^86 participants were excluded due to invalid ZIP codes and 30 due to unmatched ZCTAs, leaving 678 participants eligible for contextual linkage analysis. An additional 16 were excluded due to missing data for covariates, yielding a final analytical sample of 662 adults.

^b^Values are weighted percentages (unweighted counts shown in parentheses).

^c^Two-sided *P*-values from chi-square tests.

^d^Dominican Ethnic Concentration: derived with Location Quotients where low concentration ≤ 0.8 means underrepresentation of Dominicans; evenness 0.8–1.2 reflects parity with total population at the ZCTA level, using the entire state as the reference; higher concentration 1.2–2.0 means meaningful Dominican clustering; high concentration LQs >2.0 enclave-like conditions.

^e^Includes 22 participants who were born outside of the Dominican Republic, but does not include U.S.

^f^USDA two question screener, which assess food security for the participant or anyone living with them in the household in the past month.

Bold values indicate statistical significance.

Table 1 also shows that anxiety symptoms varied significantly across enclave categories: respondents living in low-concentration areas reported the highest mean anxiety (mean = 1.23), while those in higher-concentration areas had lower average scores (0.85 for both groups). Anxiety patterns mirrored the distribution of key sociodemographic characteristics: the enclave areas contained a disproportionate share of older adults (38.6%), lower-income households (54.4%), households with no children (61.4%), and foreign-born respondents (62.4%). In contrast, low-concentration areas were more socioeconomically advantaged, with nearly half (46.3%) earning ≥U.S.$ 80,000, a larger proportion of U.S.-born adults (63.9%), and at least one child per household (66.0%). Significant differences across enclave categories were observed for age, income, state of residence, nativity, and children in the household (all *p* < 0.01), whereas the sex distribution did not vary by enclave type (*p* = 0.81). Finally, household food insecurity was significantly less prevalent among respondents living in areas with higher Dominican ethnic concentrations; nearly 39% of individuals in low-segregation areas reported low food security compared to approximately 21%−26% in segregated and high-segregation areas (χ^2^= 4.04, *p* = 0.007).

**Figure 2:**
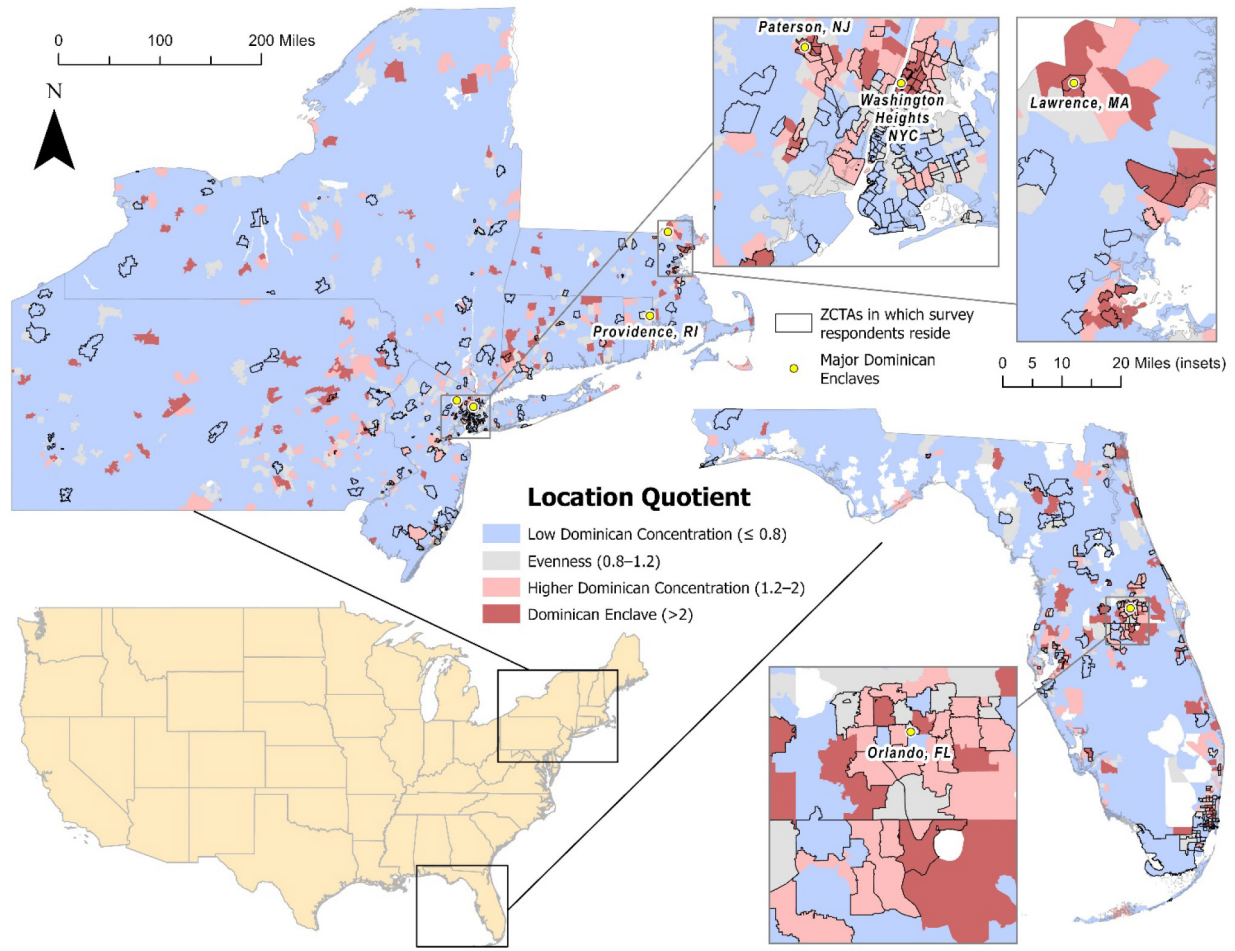
Geographic distribution of Dominican ethnic concentration across ZIP Code Tabulation Areas (ZCTAs) in the Northeastern and Southeastern United States, measured using a location quotient. Insets highlight areas with survey respondents, with shading indicating levels of Dominican segregation (low, evenness, segregation, and high segregation).

Figure 2 illustrates the geographic distribution of Dominican ethnic concentrations across ZIP Code Tabulation Areas (ZCTAs) in the Northeastern and Southeastern United States. Areas of high Dominican concentration cluster in metropolitan regions with long-standing Dominican settlements, including northern Manhattan in New York City, particularly neighborhoods such as Washington Heights and Inwood, as well as adjacent areas of the Bronx. Outside New York, elevated concentrations are evident in cities such as Lawrence and Providence, which have been documented in prior research as secondary Dominican settlement destinations. In the Southeast, high and moderate Dominican concentrations appear in South and Central Florida, including the Miami metropolitan area and inland regions surrounding Orlando, reflecting more recent migration and dispersion patterns observed among Dominican populations.

Table 2 presents the survey-weighted generalized structural equation model results examining the association between Dominican ethnic enclave concentration and anxiety symptoms, and the extent to which this association is mediated by household food insecurity using the centered log-transformed enclave measure. Overall, a higher Dominican ethnic concentration was significantly associated with lower anxiety symptoms (total effect β = −0.142, SE = 0.042, *p* = 0.001), supporting subsequent mediation testing. In the first stage (Path a), a higher log-transformed Dominican concentration was significantly associated with lower odds of experiencing low food security (β = −0. 251, SE = 0.117, *p* = 0.032). This suggests that even after compressing extreme enclave values, residents of more concentrated Dominican areas are meaningfully less likely to report food insecurity. No individual-level covariates, including age, sex, income, or nativity, were significantly related to food security, except for children living in the household.

**Table 2:** Generalized structural equation model of the association between Dominican enclave concentration (log-centered lq), food insecurity, and anxiety symptoms (*N* = 662).

Path/effect^1^,^3^,^4^	Outcome	Coefficient (β)	SE	*p*-value	95% CI
Path A: Dominican concentration (LQ, log) → food insecurity	Food insecurity^2^	−0.251	0.117	**0.032**	−0.481, −0.022
Path B: food insecurity → anxiety	Anxiety (GAD-4)	0.586	0.071	**< 0.001**	0.448, 0.725
Direct effect (c′)					
Dominican ethnic concentration (LQ) → Anxiety	Anxiety (GAD-4)	−0.076	0.040	0.057	−0.154, 0.002
Indirect effect (a × b)					
LQ → Food insecurity → Anxiety	Anxiety (GAD-4)	−0.147	0.069	**0.034**	−0.283, −0.011
Total effect (c)					
Dominican ethnic concentration (LQ) → Anxiety	Anxiety (GAD-4)	−0.223	0.080	**0.005**	−0.379, −0.067

^1^Models were estimated using survey-weighted generalized structural equation modeling to account for the complex sampling design of the 2021 CUNY Dominican Health Survey.

^2^The mediator (household food insecurity) was modeled using a Bernoulli distribution with a logit link.

^3^The outcome (GAD-4 mean anxiety score) was modeled using a Gaussian distribution with an identity link.

^4^All paths were adjusted for age, sex, household income, presence of children, state of residence, and nativity. Indirect, direct, and total effects were estimated using the delta method.

Bold values indicate statistical significance.

In Path b, household food insecurity remained a strong predictor of anxiety (β = 0.586, SE = 0.071, *p* < 0.001). Individuals reporting low food security had substantially higher GAD-4 scores than those who were food secure. In Path c′, the estimated direct association between log-transformed enclave concentration and anxiety was negative (β = −0.076, SE = 0.040) and consistent with the total association, although the 95% confidence interval included the null value (*p* = 0.055). Older age and male sex were associated with lower anxiety scores.

The mediation results suggested a statistically detectable indirect effect of household food security in the association between Dominican concentration and anxiety (indirect effect β = −0.147, 95% CI: −0.28, −0.011; *p* = 0.034), consistent with partial mediation. The magnitude of this indirect effect was modest relative to the total association which was significant and negative (β = −0.223, SE = 0.080, *p* = 0.005), indicating that a greater Dominican concentration was associated with lower anxiety symptoms.

## 4 Discussion

Using a representative sample of Dominican adults and a geographic operationalization of ethnic concentration based on location quotients, this study provides novel evidence that a greater Dominican concentration is associated with lower anxiety symptoms. Importantly, this association was partly mediated by reduced household food insecurity. These findings extend prior studies using similar geographic-based measures in a nationally representative sample of Latinos and found protective effects on mental health for men living in Latino communities (30). The broader literature on Latino concentration and mental health remains mixed, with some studies using residential segregation indices have reported elevated risk of anxiety disorders in Latino-dense neighborhoods with attenuation after accounting for nativity, generation, or individual-level socioeconomic factors (56–58). As others have noted, these inconsistencies likely reflect heterogeneity in Latino subgroup histories, migration pathways, and community infrastructures, which shape the social meaning and functional characteristics of enclaves (25, 30). By focusing exclusively on Dominicans, our study addresses an important gap and underscores the need for subgroup-specific analyses rather than pan-ethnic generalizations.

Our mediation analysis advances the literature by demonstrating that the protective association between Dominican concentration and anxiety operates, in part, through reduced household food insecurity. While the adverse relationship between food insecurity and mental health is well established, the mechanisms linking food insecurity to anxiety remain contested (59). Emerging evidence suggests that food insecurity may exert its influence not only through nutritional pathways, such as caloric insufficiency or micronutrient deficiencies, but also through psychosocial processes, including chronic stress, uncertainty, and disruptions to culturally or socially meaningful food practices (20). In this context, the experience of food insecurity itself—including the inability to procure preferred foods, adhere to food-related norms, or participate in shared meals—may independently erode emotional wellbeing. Our study highlights the need to further test whether place-based factors are central to the psychosocial pathway, in which food and nutrition serve as anchors of cultural continuity, social connectivity, and emotional regulation (6, 38, 39). Evidence from high-risk immigrants shows that when culturally familiar foods are accessible, there is improved mental health status reflected in the ability of immigrants to share their cultures and identity while rebuilding their social networks and enhancing a sense of belonging (19). Yet much of the empirical literature on food security among Latinos has focused on Mexican-origin populations (16, 36, 60–63), leaving Caribbean Latino groups comparatively understudied. One rare study comparing Puerto Rican and Dominican households found that Dominican households reported higher levels of food security and more consistent access to balanced meals, despite similar levels of poverty and Puerto Ricans' formal advantages related to U.S. citizenship and their longer incorporation into U.S. labor markets (42). More recent evidence from the SOL Youth study suggests that food security among Dominican and other Hispanic/Latino households may be sustained through adaptive food shopping strategies and reliance on ethnic food environments, even in the context of high food costs and economic constraint (64, 65).

Ethnographic and historical scholarship provides further insight into how food environments in areas with a high concentration of Dominicans may buffer against food insecurity. Research among Caribbean Latinos, including Dominicans, shows that individuals remain in—or remain socially connected to—neighborhoods where staple foods are readily available, with *bodegas*, ethnic groceries, and supermarkets playing a central role in everyday food procurement (5). Beyond access to ingredients, these establishments often function as social institutions that provide informal support during hardships. Kaufman and Karpati (66) work in New York City documents how *bodegas* have historically extended informal credit to food-insecure households, illustrating how food environments can mitigate material scarcity through embedded social relations (66). Complementary quantitative evidence from broader Latino samples, including Dominicans, shows that Spanish-speaking households are more likely to rely on ethnic food stores within distinct shopping typologies (65). These establishments are known to provide immigrant Latinos with cheap, culturally relevant ingredients to recreate traditional dishes that are not only nutritious (5, 21) but also help immigrants “produce a new sense of place and belonging” in their new homes (45, 46). These cooking practices bridge food environments and household wellbeing, and prior ethnographic work among Dominican women highlights cooking as a form of self-empowerment, care, and healing (67). This work aligns with broader scholarship framing cooking as a practice that merges nourishment with social meaning and relational labor (68, 69). Furthermore, the preparation of culturally recognized dishes, such as *mangú*, illustrates how basic, affordable staples can be transformed into powerful cultural indices of Dominicanness in New York City (67). As Marte documents, such dishes are often shaped by past experiences of food insecurity and are valued for their ability to “stretch,” feed many people quickly, and uphold moral commitments to family and community. Taken together, these qualitative findings highlight how households are buffered against food insecurity through informal food sharing, ethnic food retail density, and culturally embedded social support networks that foster social connectedness. Moreover, this work highlights specific ways in which Latino immigrant neighborhoods support healthy diets, as found in other studies, especially among immigrants (25, 34).

This study has several limitations. First, the cross-sectional design precludes causal inference and limits our ability to assess how neighborhood changes over time shape food security and mental health trajectories, as others have noted (11). For example, we cannot rule out selective processes over time, whereby individuals with lower anxiety or stronger social ties may be more likely to reside in or remain in areas with a high concentration of Dominicans (29). Second, our use of LQ captures relative concentration rather than multidimensional residential segregation, which encompasses isolation, evenness, and clustering dynamics not fully reflected in LQs (70–72). Like all spatial measures, LQs are also subject to the modifiable areal unit problem (i.e., how data can be grouped into arbitrary areas (units) at different scales from the same underlying data to provide different results), which may cause statistical bias (73). Third, the absence of data on dietary intake/quality prevented us from testing the nutritional mechanisms linking food insecurity to anxiety, including pathways involving micronutrient deficiencies, inflammation, gut–brain interactions, and stress physiology (74–77). Finally, neighborhood enclaves may simultaneously promote health through diet while constraining other domains such as physical activity, underscoring the need for integrative designs capable of capturing trade-offs across health behaviors (25, 27).

Despite these limitations, our findings provide evidence that Dominican enclaves are associated with lower anxiety and that this protective effect is conferred, in part, through reduced food insecurity, even in the context of socioeconomic disadvantage. Prior ethnographic work points to the rich traditions of cooking, food sharing, and reliance on ethnic food retailers that likely constitute unmeasured protective resources. Future research should explicitly measure these practices, incorporate longitudinal and mixed-methods designs, and move beyond deficit-oriented assumptions that equate food insecurity with a lack of skills or agency, given the evidence to the contrary (78). A more nuanced attention to food prices, ownership of food stores, cultural relevance, social embeddedness of food environments, and social connectivity promoted by these environments may be critical for both study design and intervention development (21, 55). As Gabaccia (4) observed, ethnic businesses make “culinary conservatism possible,” but their broader role in fostering community cohesion and psychological resilience remains understudied (4). Understanding how food access, stress, and identity intersect across diverse foodscapes is essential for advancing theories of dietary acculturation, which are still largely linear, and for designing equitable, culturally grounded public health interventions among Dominicans and other Latino subgroups.

These findings also carry implications for health policy and practice. Policies aimed at improving food security among immigrant populations often prioritize increasing the quantity of food assistance but rarely account for the cultural and social infrastructures that shape how food is accessed and shared within migrant communities. Supporting neighborhood-level food environments that sustain culturally relevant foods—such as immigrant-owned food retailers and local ethnic food supply chains—may represent an important yet under recognized strategy for promoting both nutrition security and mental wellbeing among immigrant populations. This consideration may be particularly relevant for Dominican communities, who have emerged as prominent entrepreneurs in the urban food retail sector in New York City and other U.S. metropolitan areas, operating extensive networks of bodegas, supermarkets, and food distribution businesses that play a central role in neighborhood food access. Clinicians and public health practitioners working with Dominican and other Latino communities may also benefit from recognizing the role that culturally meaningful food practices and social food networks play in buffering stress and supporting mental health.

## Data Availability

The raw data supporting the conclusions of this article will be made available by the authors, without undue reservation.
